# Form and Function: A Study on the Distribution of the Inflectional Endings in Italian Nouns and Adjectives

**DOI:** 10.3389/fpsyg.2021.720228

**Published:** 2021-10-07

**Authors:** Valentina Nicole Pescuma, Chiara Zanini, Davide Crepaldi, Francesca Franzon

**Affiliations:** ^1^Neuroscience Area, International School for Advanced Studies, Trieste, Italy; ^2^Romanisches Seminar, University of Zurich, Zurich, Switzerland

**Keywords:** grammatical gender, grammatical number, adjective inflection, noun inflection, declensional classes, inflectional morphology, language resource, contextual and inherent inflection

## Abstract

Inflectional values, such as singular and plural, sustain agreement relations between constituents in sentences, allowing sentence parsing and prediction in online processing. Ideally, these processes would be facilitated by a consistent and transparent correspondence between the inflectional values and their form: for example, the value of plural should always be expressed by the same ending, and that ending should only express plural. Experimental research reports higher processing costs in the presence of a non-transparent relation between forms and values. While this effect was found in several languages, and typological research shows that consistency is far from common in morphological paradigms, it is still somewhat difficult to precisely quantify the transparency degree of the inflected forms. Furthermore, to date, no accounts have quantified the transparency in inflection with regard to the declensional classes and the extent to which it is expressed across different parts of speech, depending on whether these act as controllers of the agreement (e.g., nouns) or as targets (e.g., adjectives). We present a case study on Italian, a language that marks gender and number features in nouns and adjectives. This work provides measures of the distribution of forms in the noun and adjective inflection in Italian, and quantifies the degree of form-value transparency with respect to inflectional endings and declensional classes. In order to obtain these measures, we built Flex It, a dedicated large-scale database of inflectional morphology of Italian, and made it available, in order to sustain further theoretical and empirical research.

## 1. Introduction

Languages can express grammatical features through inflectional morphology. For instance, in English the singular and plural values of the grammatical feature of number can be expressed through the forms *apple* (SG, singular) and *apples* (PL, plural), whereby the plural form is realized through the ending *-s*. On the language processing side, the relevance of the role of inflectional features for comprehension is attested, for instance, by the ability to pick up inflectional regularities from the first stages of language development shown by children as young as 12 months (Ferry et al., 2020). One might expect these processes to be enabled and facilitated by consistency in the correspondence between an inflectional feature's value and a word form. For example, the value of plural should ideally always be expressed by the same affix in a certain language (for a review: Huettig et al., 2011). In line with this account, transparency does appear to facilitate the acquisition of inflectional features, as shown in a recent study with Bulgarian- and Russian-speaking children on the acquisition of grammatical gender (Ivanova-Sullivan and Sekerina, 2019). Similarly, it has been noted that, in sentence comprehension, speakers of morphologically rich languages (like Italian or German) are more likely to use inflectional cues than speakers of languages having highly constrained word order (like English; Bates et al., 1982; MacWhinney et al., 1984; MacWhinney and Bates, 1989) and that in second language acquisition less proficient speakers are more likely to rely on ending cues than more proficient speakers and, as a result, are faster and more accurate in retrieving the gender of nouns whose endings transparently convey the corresponding morphological value (e.g., for German-English bilinguals: Bordag et al., 2006; for Basque-Spanish bilinguals: Caffarra et al., 2017). Unsurprisingly, a facilitation in the processing of grammatical gender information when the relation between ending and value is transparent or regular has been observed in a wealth of studies, comprising behavioral paradigms (e.g., Bates et al., 1995, 1996; Taft and Meunier, 1998; Gollan and Frost, 2001; De Martino et al., 2011), electrophysiological (e.g., Caffarra et al., 2015) and neural evidence (e.g., Miceli et al., 2002; Russo et al., 2021), including studies on aphasia and semantic dementia (Luzzatti and De Bleser, 1996; Lambon Ralph et al., 2011; Franzon et al., 2013).

### 1.1. The Form-Function Inconsistency Issue

However, consistency is not always observed in the inflectional paradigms of natural languages (Corbett, 2006). In fact, a lack of transparency between forms and feature values is more the rule than the exception (e.g., one value expressed through several different endings, different values expressed through the same ending, or values apparently expressed by no ending). For example, in English the plural value is not always conveyed by the final *-s*. Cases of allomorphy like *ox*/*oxen*, suppletivism like *child*/*children*, and apophony like *foot*/*feet* are not infrequent, such that the very Bloomfieldian notion of morphemes as the smallest linguistic units bearing meaning (Bloomfield, 1933) has been questioned (Matthews, 1974; Anderson, 1992; Aronoff, 1994; Baayen et al., 2011). Although these forms can be seen as sub-regularities (due to the fact that they are fossils of grammatical rules that are no longer active in a synchronic perspective, and therefore no longer productive; e.g., Anderson, 1992), they are nonetheless “irregular” since to say that a form has a regular inflection is to say that it has the inflection one would expect unless one knew that it was different (Matthews, 1991, p. 130).

Some accounts suggest that the presence of irregular inflectional paradigms, which may initially yield errors related to an over-generalization of regular patterns, ultimately supports learning processes (Ramscar et al., 2018). Furthermore, as noted in relation to verb inflection by Marzi et al. (2019), while one would expect maximal contrast between forms to yield immediate discrimination and recognition of inflectional values, this has a cost in terms of the storage space required for too many different forms. The coexistence of regular and irregular forms within the language has indeed been ascribed to an inevitable trade-off between maximal discriminability, on the one hand, and a degree of regularity sufficient to allow successful generalization, on the other (Blevins et al., 2017). A way in which this relation between ambiguity and informativeness of inflectional systems has been operationalized is the implementation of entropy metrics (Dye et al., 2017; Mickus et al., 2019; Williams et al., 2020; Franzon and Zanini, 2021) as defined by Shannon, 1948,^1^. In this sense, entropy allows to quantify the probability for a feature to be associated to one or more given forms, and vice versa, assessing the consistency of this association.

### 1.2. Noun and Adjective Forms in Italian

The inflectional system of Italian nouns and adjectives comprises four combinations of inflectional values (i.e., masculine singular, masculine plural, feminine singular, and feminine plural). However, noun inflection in Italian has hardly been investigated (Franzon and Zanini, 2021), and an account of adjective inflection in Italian is completely missing, up to date. Furthermore, although the reason why form-value inconsistency occurs for inflectional features is still debated, form-value inconsistency in Italian inflection has hardly ever been quantified in these terms. Given that the notion of transparency is pivotal for psycholinguistic accounts describing the architecture of the mental lexicon (Crepaldi et al., 2010; Davis and Rastle, 2010; Amenta and Crepaldi, 2012; Marelli et al., 2015; Milin et al., 2017; Marelli and Amenta, 2018), measures of the transparency of the inflectional systems can significantly contribute to the understanding of how words are processed both in isolation and in sentence contexts. In the present study, a first step is taken to assess the extent to which inflectional forms consistently represent a given value in Italian. We will assess how the inflected forms of nouns and adjectives are distributed within the finite set of the combinations of feature values of gender and number.

Indeed, Italian nouns and adjectives are necessarily inflected for number (singular vs. plural) and gender (masculine vs. feminine), whose values are both expressed in a single fusive ending [e.g., *gatto* “cat(M).SG,” *gatta* “cat(F).SG,” *gatti* “cat(M).PL,” *gatte* “cat(F).PL”]. Crucially, the endings of Italian nouns and adjectives cannot be considered unambiguous formal cues for gender and number values; nonetheless, the correspondence between forms and functions displays some recurrent patterns. Nouns have traditionally been divided into declensional classes according to the inflectional endings of their singular and plural forms. Considering a declensional class as a set of lexemes whose members each select the same set of inflectional realizations (Aronoff, 1994, p. 64), six declensional classes have been described for nouns in Italian (Iacobini and Thornton, 2016, p. 195): Class I (SG: *-o*; PL: *-i, libro - libri* “book - books”); Class II (SG: *-a*; PL: *-e, rosa - rose* “rose - roses”); Class III (SG: *-e*; PL: *-i, fiore - fiori* “flower - flowers”); Class IV (SG: *-a*; PL: *-i, problema - problemi* “problem - problems”); Class V (SG: *-o*; PL: *-a, uovo - uova* “egg - eggs”); Class VI (invariable nouns, various endings: e.g., *re* “king / kings”). For adjectives, five declensional classes have been identified (Iacobini and Thornton, 2016, p. 204): Class I (M.SG: *-o*; M.PL: *-i*; F.SG: *-a*; F.PL: *-e, bello - belli - bella - belle* “beautiful”); Class II (M.SG and F.SG: *-e*; M.PL and F.PL: *-i, grande - grandi* “big”); Class III (M.SG and F.SG: *-a*; M.PL: *-i*; F.PL: *-e, belga - belgi - belghe* “Belgian”); Class IV (M.SG and F.PL: *-e*; M.PL: *-i*; F.SG: *-a, sornione - sornioni - sorniona* “seemingly friendly”); Class V (invariable adjectives, various endings: e.g., *blu* “blue”).

Noun Classes I and II are quite transparent with respect to gender features (comprising, respectively, mostly feminine and mostly masculine nouns), and so is adjective Class I. However, there is no straightforward correspondence between declensional classes and gender features. This entails that, considering the whole declensional system, no ending is unambiguously related to one value, and likewise no value is unambiguously related to one ending. This is possibly due to the fact that Italian, unlike languages such as English or Spanish, has a non-additive, non-sigmatic plural and, in general, its words must end with a vowel. As such, Italian noun and adjective forms are distributed in a narrow space subtended by just four vowels: *-o, -a, -e, -i*. In principle, a speaker exposed to a novel noun ending in *-e*, in the absence of other cues (such as an article or any other determiner), would not be able to disentangle whether the noun is a feminine plural of the first class like *sedie* “chairs,” a feminine singular of the third class like *tigre* “tiger,” or a masculine singular of the third class like *elefante* “elephant.” Similarly, the masculine singular value is realized with different endings, such as *-o* (*divano* “couch”) and *-e* (*elefante* “elephant”), *-a* (*problema* “problem”).

We are aware that in our experience as speakers and readers we are hardly exposed to nouns in isolation. Therefore, a transparent form-value relation may not be a necessary nor a sufficient cue to sustain learning processes. Indeed, inflection plays a functional role in establishing morpho-syntactic agreement (e.g., *the apple*.SG *is*.SG *red* vs. *the apples*.PL *are*.PL *red*). Agreement can be described as the systematic covariance between a semantic or formal property of one element and a formal property of another (Steele, 1978, p. 610). It has been noted that agreement involving inflectional features, such as the feature of number with its singular and plural values, allows to disambiguate the relations between words in sentence parsing, reducing processing effort by favoring word predictions (Wicha et al., 2004; Huettig et al., 2011; Dye et al., 2017). More precisely, nouns are generally the “controllers,” i.e., the elements that determine the agreement and whose expression of agreement features is usually covert. On the other hand, adjectives (as well as other functional elements such as articles) are “targets,” i.e., the elements whose form is determined by the controllers (Corbett, 2006). In turn, this relates to another related aspect, that is, the difference between inherent and contextual inflection proposed in theoretical linguistics accounts (Booij, 1993, 1996; Di Domenico, 1997), and seldom explored experimentally (De Vincenzi and Di Domenico, 1999; Franzon et al., 2014). Inherent and contextual inflection are here exemplified, respectively, by nouns, which have an inherent, context-autonomous gender, and determine the form of other parts of speech, and adjectives whose gender and number will be determined by those of the noun they are related to. As we will discuss in section 4.1.3, this entails interesting differences in the distribution of inflectional features of Italian nouns and adjectives. It follows that less variability is expected in the target, i.e., the adjective forms having contextual inflection, since gender and number play here a merely functional, context-driven role and, as such, on the computational side, can serve more for prediction purposes allowing a maximal discriminability between gender and number values. A new metric therefore appears more suitable to quantify form-value consistency, while moving away from binary, categorical and non-quantifiable distinctions such as “transparent vs. opaque” or “regular vs. irregular.”

### 1.3. Objectives of the Study

In Italian, studies concerning nominal inflection or nominal agreement have often relied on the morphological competence of the experimenters in controlling the transparency of the stimuli selection, even when the processing of inflected word forms was a central part of the study (Luzzatti and De Bleser, 1996; Caffarra et al., 2015; Franzon et al., 2016; Arcara et al., 2019; Zanini et al., 2020). This shortcoming has been likely due to the long-standing unavailability of suitable linguistic resources to measure noun transparency. To our knowledge, a resource for nominal inflection in Italian was released only recently: the database DeGNI (De Martino et al., 2019), which is based on the Colfis corpus (Bertinetto et al., 2005), containing type frequency information for mostly singular forms. Token frequency information, which is considered a better estimate of actual language use, is not provided.

The present work aims at providing an account of the distributional properties of noun and adjective inflection in Italian, to quantify the degree of form-value transparency and to investigate the distribution of forms across inflectional values and declensional classes. In order to compute such metrics, we built a dedicate large scale resource: Flex It, a database of inflectional morphology of Italian, which will be described in section 2. Flex It is set available as a freely usable resource, with the aim to enable further empirical and theoretical research.

### 1.4. Definition of the Terms Used in the Study

Before moving to a more thorough description of the Flex It database, it is worth summarizing and defining a few terms used in this paper (especially in the light of inconsistent terminology in the literature): **word form**, any inflected word (e.g., *gatti* “cats,” is the Italian plural form of the noun *gatto* “cat”); **ending**, the inflectional termination of a word (e.g., *-i* in the Italian noun *gatti* “cats”); **declensional class**, set of lexemes whose members each select the same set of inflectional realizations (e.g., the Italian nouns *gatto* “cat,” and *cane* “dog,” belong to two different declensional classes since they do not share the same endings: *gatt-o/-i* “cat/cats,” vs. *can-e/-i* “dog/dogs”); **feature**, any grammatical characteristic/property for which a word can be specified (e.g., Italian nouns can be specified for number: *gatto* “cat,” vs. *gatti* “cats”); **value**, any possible specification of a given feature (e.g., in Italian, the feature of number has two values: singular and plural); **token**, the total number of occurrences of a word form in the database (e.g., the plural word form *gatti* “cats,” occurs N times); **type**, every different type of word form in the database, regardless of its total number of occurrences (e.g., even if the plural word form *gatti* “cats,” occurs N times, it is counted only once).

## 2. Methods

### 2.1. The Flex It Database

In building Flex It, our goal was to gather data for the present study, as well as to provide a large-scale morphologically annotated database and set it available for further research. The database and its descriptive analyses were developed using R (R Core Team, 2021) and can be downloaded from: https://github.com/franfranz/Flex_it.

The database contains the token frequencies of 71,954 Italian word forms (33,637 noun types and 38,317 adjective types), annotated for inflectional ending, gender, number, declensional class, lemma, grade of adjectives, raw and standardized measures of frequency. We obtained token frequency measures from ItWaC, the largest freely available corpus of Italian, consisting of 1.9 billion tokens from web-collected texts (Baroni et al., 2009). While the size and text variety of this corpus suffice in providing an excellently representative sample of language use, its morphological tagging is at the part of speech (POS) level. A finer-grained morphological annotation, comprising also the indication of gender (feminine - masculine) and number (singular - plural) feature values for adjective and noun types, was retrieved from Morph-it!, a list containing approximately 500,000 word forms, tagged for lemma (Zanchetta and Baroni, 2005).

The Flex It database provides morphological information on a wide scale: besides tags for gender, number and for inflectional endings, we reported a tag for inflectional class. As stated in section 1, in Italian, the inflectional ending corresponds to the last phoneme of a word form, in the noun as well as in adjective declension. In written text, it will in turn correspond to the last letter, due to the orthographically transparent writing system. In order to obtain the inflectional ending, the last character of each word form was stripped. Inflectional paradigms were reconstructed by coupling the endings occurring for the same lemma. Embracing the inherent *vs*. contextual theoretical distinction (as discussed in section 1), inflectional paradigms for nouns include the number values as a two-cell paradigm, whereas inflectional paradigms of adjectives include gender and number values as a four-cell paradigm (In other words, the lemma of a noun is lexically specified for gender, can be inflected in the singular or in the plural, and thus can assume two combinations of values. Instead, the form of an adjective is determined by the values of the noun it modifies and, thus, each lemma can assume four combinations of values). In some cases, only one form was attested for a lemma; in this case, a “NA” tag was assigned in place of the ending not attested in our database even if supposed from a theoretical point of view. In the case of identical word forms for the singular and the plural, an “Inv” tag signals the invariance. In order to avoid some possible confounds derived from the tagging of the original resources, invariance and other phenomena that lead to the presence of ambiguous forms had to be tackled before quantifying the morphological transparency of inflectional classes and exponents.

### 2.2. Ambiguous Forms

Some noun types are homograph to other POS, such as *apparecchio* noun(M).SG “device,” or verb-I.SG.PRES “I prepare.” These cases were not problematic for the database, as we collected the token frequency for the occurrences of words tagged as nouns in the ItWaC corpus. The same method was applied to the collection of adjective types homograph to types tagged as other POS. Similarly, in word forms occurring as nouns as well as adjectives, such as *manifesto* noun(M).SG “poster,” or adj(M).SG “evident,” or *sole* noun(M).SG “sun,” or adj(F).PL “alone,” the token frequency measures reported in the noun and in the adjective lists refer to the occurrences, respectively, tagged as nouns and as adjectives in the corpus. Since ItWaC is tagged at the POS level, no confounds should occur in measures taken on homograph forms belonging to different POS.

Nevertheless, some types sharing the same POS inflected in different feature values do surface with an identical word form. This can be due to several factors. In cases like *latte* noun(M).SG “milk”/noun(F).PL “tin cans,” the difference in meaning undoubtedly points to two different lemmas incidentally surfacing in a homograph form. In other cases, homography is observed in semantically related words and has a more systematic aspect due to the intersection of inflectional classes, as in *cameriere* noun(M).SG “waiter,” and noun(F).PL “waitresses”; here, the singular masculine in the *e_i* class is confounded with the plural feminine in the *a_e* class. Similarly, other types surface in the same word form in the singular, like *musicista* noun(M).SG, noun(F).SG “(male/female) musician,” showing different forms in the plural, respectively, feminine, *musiciste*(F).PL “female musicians,” and masculine, *musicisti*(M).PL “male musicians.” Other types are identical in the singular and in the plural: this lack of change of form will be the hallmark of an “invariant” inflectional class. Finally, some nouns, mostly denoting humans (*portavoce*) “spokesperson,” show the same form for all four features. We collected all the ambiguous forms, independently of the factors that determine their ambiguity. Ambiguous forms make up the 0.056 (in proportion) of the total noun list, and adjectives make up the 0.169 (in proportion) of the adjective list.

In Tables 1, 2, we report the number of ambiguous forms for nouns and adjectives, respectively, and their occurrence across the inflectional features. A form is reported as an example for each kind of ambiguity.

**Table 1:** Number of ambiguous noun forms.

**Feature values**		**N.forms**
F. SG. - F. PL. - M. SG. - M. PL.	*portavoce* “(fe)male spokesperson(s)”	43
F. SG. - F. PL. - M. SG.	*radio* “radio - radios - radius bone”	11
F. PL. - M. SG. - M. PL.	*marine* “marinas - mariner - mariners”	6
F. SG. - M. SG. - M. PL.	*boa* “buoy - boa - boas”	9
F. SG. - F. PL.	*analisi* “analysis - analyses”	246
F. PL. - M. PL.	*abitanti* “female residents - male residents”	190
F. PL. - M. SG.	*cameriere* “waitresses - waiter”	34
F. SG. - M. PL.	*sequestri* “kidnapping unit - requisitions”	1
F. SG. - M. SG.	*abitante* “female resident - male resident”	240
M. SG. - M. PL.	*quiz* “quiz - quiz”	969
Total		1,749

**Table 2:** Number of ambiguous adjective forms.

**Feature values**		**N.forms**
F. SG. - F. PL. - M. SG. - M. PL.	*antidroga* “antidrug”	268
F. SG. - F. PL. - M. SG.	*molle* “soft”	9
F. PL. - M. PL.	*abili* “skilled”	2,451
F. SG. - M. SG.	*abile* “skilled”	2,663
Total		5,391

For each of the forms ambiguously surfacing in more than one combination of feature values, it is possible to retrieve its type frequency, due to the tag provided by the Morph-it! list. However, the token frequency for each of these types cannot be disambiguated into the different values. For example, it is not possible to state how many of the 5,232 tokens of the word form *cameriere* are occurrences of the type noun(M).SG “waiter” and how many of the type noun(F).PL “waitresses.” In order to avoid this potential confound, we considered the type frequencies of ambiguous nouns in our analysis, but we limited our counts on the token frequency of non-ambiguous forms.

## 3. Results

We measured the distribution of Italian nouns and adjectives in the Flex It database to assess the entropy of the morphological systems with respect to the features of gender and number. To this end, we considered the distribution of the word forms from two different points of view: (i) first, the arrangement of the word forms according to each declensional class (e.g., the amount of word forms that belong to Class I, sharing the same endings *o_i* for the singular and the plural, and convey the value of masculine vs. the amount of word forms that belong to Class I and instead convey the value of feminine; section 3.1.1 for nouns and section 3.2.1 for adjectives); (ii) second, the distribution of the word forms across all possible combinations of values (F.SG, F.PL, M.SG, M.PL) with respect to each inflectional ending (e.g., the amount of word forms in *-o* that convey the value combination of masculine singular vs. the amount of word forms in *-o* that instead convey the value combinations of masculine plural or feminine singular or feminine plural; section 3.1.2 for nouns and section 3.2.2 for adjectives).

### 3.1. Noun Inflection

#### 3.1.1. Declensional Classes

The number of type and tokens for each declensional class are reported in Table 3. The invariant nouns are grouped together as a single class “Inv.” The “Other” tag in the table collects the nouns that would be expected to be invariant but are attested as inflected in some cases, as *sport*(M).SG/*sports*(M).PL - *corpus*(M).SG/*corpora*(M).PL. For each of the declensional classes, we report an entropy value *H*, calculated in the way indicated by Shannon (1948), based on the probability for each set of endings to realize the feminine or the masculine forms. In this sense, entropy is a measure of consistency in the association of a declensional class with a gender value. Low entropy values correspond to a more stable association between a declensional class and a gender value.

**Table 3:** Distribution of noun lemmas across the declensional classes (types - token).

	**Noun types**				**Noun tokens**			
**Class**	**F**.	**M**.	**Total**	**H**	**F**.	**M**.	**Total**	**H**
o_i	2	11,957	11,959	0.0023	442,454	128,733,286	129,175,740	0.033
a_e	8,318	0	8,318	0	81,295,823	0	81,295,823	0
e_i	3,268	3,907	7,175	0.9943	47,533,225	26,577,783	74,111,008	0.9415
a_i	4	932	936	0.0398	207,807	5,900,697	6,108,504	0.2142
o_a	23	23	46	1	405,856	472,125	877,981	0.9959
o_a_i[*]	14	42	56	0.8113	136,379	684,209	820,588	-
Inv	2	40	42	0.2761	893	9,004	9,897	0.4372
Other	55	89	144	0.9594	233,392	979,046	1,212,438	0.7066

[*] In Italian, a handful of nouns can have one form for the singular, but two forms for the plural (e.g., muro(M).SG “wall,” muri(M).PL “walls,” mura(F).PL “city wall”). These forms are listed as the same lemma in the resources used for the compilation of Flex It. The count for these forms are reported here for informative purposes only. It is not among the scopes of the present paper to describe these plural forms (e.g., muri(M).PL “walls” and mura(F).PL “city wall”) as resulting from the inflection of the same lexeme or two distinct, although homophonous, lexemes (each linked to a diverse plural form). For theoretical accounts concerning these forms, see (Acquaviva, 2002, 2008; Thornton, 2013).

#### 3.1.2. Inflectional Endings

**Figure 1:**
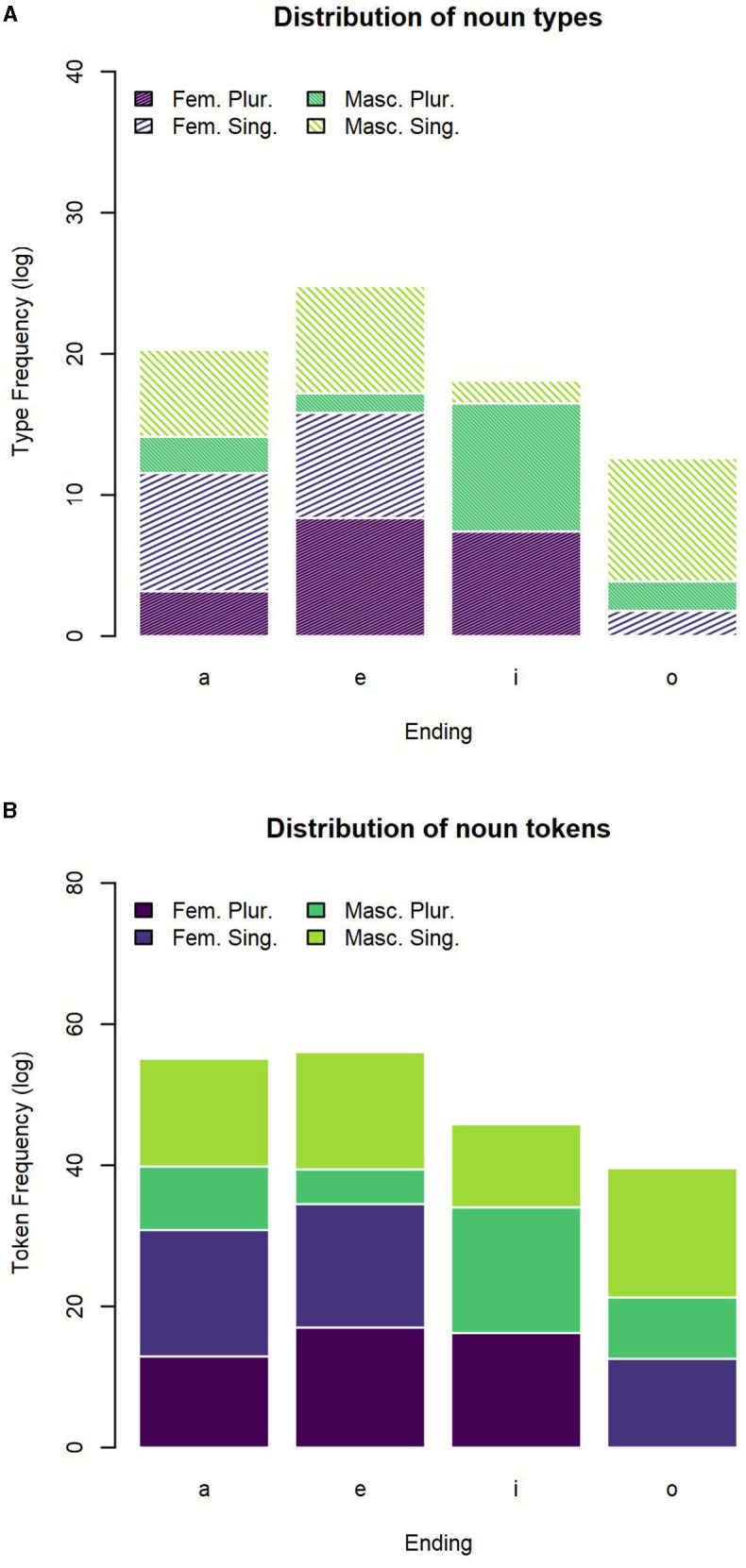
Distribution of nouns across the most frequent inflectional endings. **(A)** Number of noun types for the most frequent inflectional endings. **(B)** Number of noun tokens for the most frequent inflectional endings.

The distribution of noun types across the four most frequent inflectional endings *-a, -e, -i, -o* is reported in Table 4 and plotted in Figure 1A. The distribution of noun tokens across the four most frequent inflectional endings is reported in Table 5 and plotted in Figure 1B.

**Table 4:** Number of noun types for the most frequent inflectional endings.

**Nouns - types**						
**Ending**	**F.PL**	**F.SG**	**M.PL**	**M.SG**	**Total**	***H***
-a	23	4,331	12	477	4,843	0.5316
	(0.0047)	(0.8943)	(0.0025)	(0.0985)		
-e	4,286	1,715	3	2,002	8,006	1.4631
	(0.5353)	(0.2142)	(0.0004)	(0.2501)		
-i	1,667	0	8,661	4	10,332	0.6424
	(0.1613)	(0)	(0.8383)	(0.0004)		
-o	0	5	7	6,205	6,217	0.0221
	(0)	(0.0008)	(0.0011)	(0.9981)		

*The first column lists the endings, the second to fifth report the counts and probabilities (the proportional values in brackets) for each combination of features. In the last, we report the entropy for the ending indicated in the row*.

**Table 5:** Number of noun tokens for the most frequent inflectional endings.

**Nouns - tokens**						
**Ending**	**F.PL**	**F.SG**	**M.PL**	**M.SG**	**Total**	***H***
-a	405,856	59,533,689	8,213	4,273,650	64,221,408	0.4094
	(0.0063)	(0.9270)	(0.0001)	(0.0665)		
-e	24,251,151	39,817,093	134	16,710,610	80,778,988	1.4945
	(0.3002)	(0.4929)	(0.000)	(0.2069)		
-i	11,290,598	0	53,488,244	129,009	64,907,851	0.6868
	(0.1739)	(0)	(0.8241)	(0.0020)		
-o	0	289,291	5,963	90,593,727	90,888,981	0.0320
	(0)	(0.0032)	(0.0001)	(0.9968)		

*The first column lists the endings, the second to fifth report the counts and probabilities (the proportional values in brackets) for each combination of features. In the last, we report the entropy for the ending indicated in the row*.

We counted how many forms occur for each combination of inflectional values. Based on the probability of each ending to realize one of the possible forms, we calculated the entropy as a proxy to transparency of each of the endings. Transparency is related to a low entropy, corresponding to the fact that an ending is mostly likely to realize a specific combination of values. Such an ending will be informative of the presence of an inflectional value or combination of values. The entropy for each ending is reported in the *H* columns in the tables, respectively, calculated on the types and on the tokens.

### 3.2. Adjective Inflection

#### 3.2.1. Declensional Classes

**Figure 2:**
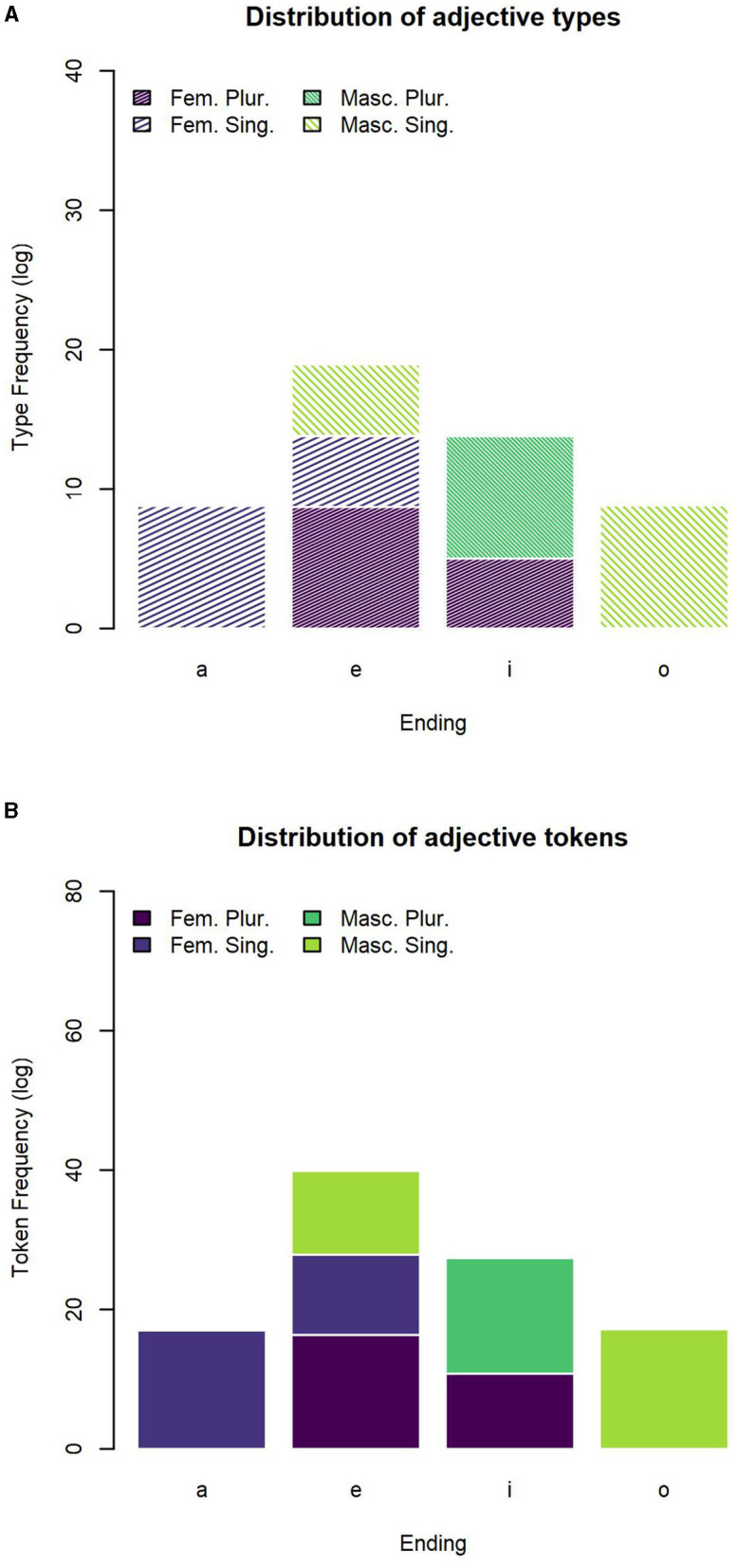
Distribution of adjectives across the most frequent inflectional endings. **(A)** Number of adjective types for the most frequent inflectional endings. **(B)** Number of adjective tokens for the most frequent inflectional endings.

The number of type and tokens for each declensional class are reported in Tables 6, 7. We find a consistent representation of the first and second declensional classes predicted by theoretical descriptions. Due to the less precise representation of adjectives in the corpus, possibly related to their lower frequency of occurrence (as shown in Figures 1, 2), we reported several defective types for which some inflected forms are not present in the corpus. In this regard, it is worth noticing that not all the possible forms of an adjective lemma predicted on a theoretical basis occur in our database (for example, an adjective lemma that can be inflected in all combinations of values, i.e., F.SG, F.PL, M.SG, and M.PL, is attested only in the F.SG). Moreover, only the first two declensional classes (which are also the most represented) include adjective forms per all possible combinations of values. Hence, the lack of occurrences of some forms in the database, instead assumed at a theoretical level, explains the apparent discrepancy between the number of declensional classes identified in the literature (i.e., five) and the number of rows in Tables 6, 7 (i.e., 12). For each of the declensional classes, we report an entropy value, which refers to the probability for each set of endings to realize the combination of feminine plural, feminine singular, masculine plural or masculine singular values. These declensional classes stem from the realization of an inflected adjective lemma. For example, the first class collected the lemmas whose occurrences end in *-a* in the feminine singular, in *-e* in the feminine plural, in *-o* in the masculine singular and in *-i* in the masculine plural. In this case, the transparency of the forms is evident in the column “Class” of Tables 6, 7, which lists four different forms. The columns *H* represents the probability for which each of the lemmas occurs as inflected in each of the value combination.

**Table 6:** Distribution of adjective lemmas across the declensional classes (types).

**Adjectives - types per declensional class**						
**Class**	**F.PL**	**F.SG**	**M.PL**	**M.SG**	**Total**	**H**
a_e o_i	5,727	6,068	5,831	6,137	23,763	1.9994
e_i e_i	148	148	148	148	592	2
NA o_i	0	0	285	287	572	1
a_NA NA	0	458	0	0	458	0
NA o_NA	0	0	0	395	395	0
a_e NA	128	131	0	0	259	0.9999
NA_e NA	242	0	0	0	242	0
NA NA_i	0	0	240	0	240	0
NA e_i	0	0	19	19	38	1
e_NA NA	0	11	0	0	11	0
e_i NA	2	2	0	0	4	1
NA e_NA	0	0	0	2	2	0

**Table 7:** Distribution of adjective lemmas across declensional classes (tokens).

**Adjectives - tokens per declensional class**						
**Class**	**F.PL**	**F.SG**	**M.PL**	**M.SG**	**Total**	**H**
a_e o_i	12,516,462	24,500,447	15,542,053	27,661,762	80,220,724	1.9292
e_i e_i	49,146	98,955	130,777	157,807	436,685	1.8916
NA o_i	0	0	52,466	165,194	217,660	0.7968
NA_e NA	154,248	0	0	0	154,248	0
NA NA_i	0	0	117,487	0	117,487	0
NA o_NA	0	0	0	82,563	82,563	0
a_e NA	35,220	47,231	0	0	82,451	0.9846
a_NA NA	0	27,666	0	0	27,666	0
NA e_i	0	0	1,891	2,530	4,421	0.9849
NA e_NA	0	0	0	2,167	2,167	0
e_i NA	122	483	0	0	605	0.7252
e_NA NA	0	132	0	0	132	0

#### 3.2.2. Inflectional Endings

The distribution of adjective types across the four most frequent inflectional endings *-a, -e, -i, -o* is reported in Table 8 and plotted in Figure 2A. The distribution of adjective tokens across the four most frequent inflectional endings is reported in Table 9 and plotted in Figure 2B. We counted how many forms occur for each combination of inflectional values. Based on the probability of each ending to realize one of the possible forms, we calculated the transparency of each of the endings. Transparency is related to a low entropy, corresponding to the fact that an ending is mostly likely to realize a specific combination of values; such ending will be informative of the presence of an inflectional value or combination of values.

**Table 8:** Number of adjective types for the most frequent inflectional endings.

**Adjectives - types**						
**Ending**	**F.PL**	**F.SG**	**M.PL**	**M.SG**	**Total**	**H**
-a	0	6,657	0	0	6,657	0
	(0)	(1)	(0)	(0)		
-e	6,097	161	0	169	6,427	0.3434
	(0.9487)	(0.0251)	(0)	(0.0263)		
-i	150	0	6,523	0	6,673	0.1551
	(0.0225)	(0)	(0.9775)	(0)		
-o	0	0	0	6,819	6,819	0
	(0)	(0)	(0)	(1)		

*The first column lists the endings, the second to fifth report the counts and probabilities (the proportional values in brackets) for each combination of features. In the last, we report the entropy for the ending indicated in the row*.

**Table 9:** Number of adjective tokens for the most frequent inflectional endings.

**Adjectives - tokens**						
**Ending**	**F.PL**	**F.SG**	**M.PL**	**M.SG**	**Total**	**H**
-a	0	24,575,344	0	0	24,575,344	0
	(0)	(1)	(0)	(0)		
-e	12,705,930	99,570	0	162,504	12,968,004	0.1620
	(0.9798)	(0.0077)	(0)	(0.0125)		
-i	49,268	0	15,844,674	0	15,893,942	0.0303
	(0.0031)	(0)	(0.9969)	(0)		
-o	0	0	0	27,909,519	27,909,519	0
	(0)	(0)	(0)	(1)		

*The first column lists the endings, the second to fifth report the counts and probabilities (the proportional values in brackets) for each combination of features. In the last, we report the entropy for the ending indicated in the row*.

## 4. Discussion

For the first time, we measured the distribution of Italian nouns and adjectives across the feature values for which they can be specified to assess the entropy of the morphological inflectional system. For this purpose, we created a large database, Flex It, combining the corpus ItWaC, the largest freely available corpus of Italian which is tagged at the part of speech level, and Morph-it!, a list of word forms comprising a finer-grained morphological annotation. Based on the probability of the inflectional endings to convey the possible feature values, we calculated the entropy as a proxy to transparency of each of the endings. More precisely, we considered the distribution of the word forms from two different points of view: (i) the distribution of the word forms across all possible combinations of values (F.SG, F.PL, M.SG, M.PL) with respect to each inflectional ending (e.g., the amount of word forms in *-o* that convey the value combination of masculine singular vs. the amount of word forms in *-o* that instead convey the other combinations of values), and (ii) the arrangement of the word forms according to each declensional class (e.g., the amount of word forms that belong to Class I, sharing the same endings *o_i* for singular and plural, and convey the value of masculine vs. the amount of word forms that belong to Class I and instead convey the value of feminine).

### 4.1. Form-Function Consistency

#### 4.1.1. Transparency of Inflectional Endings

First, we found that masculine singular nouns mostly end in *-o*, which is indeed associated to the lower close-to-min entropy of the distribution (of both types and tokens). A higher entropy is instead detected for *-a* (which mostly realizes -but it is not restricted to- feminine singular forms) and for *-i* (which mostly realizes -but it is not restricted to- masculine plural forms). The highest entropy of the distribution was spotted for *-e* which is almost equally likely to form feminine singular, feminine plural, and masculine singular nouns. Thus, the overall system seems to reflect the trade-off between maximal discriminability and maximal regularity that has been argued in the literature for other languages and other grammatical systems (as mentioned in section 1; e.g., Blevins et al. 2017).

The distribution of nouns mirrors, in broad terms, that of adjectives, albeit with some non-negligible differences. Indeed, we observed much less variability in the distribution of adjective forms across feature values in comparison to nouns. In this case, the endings *-a* and *-o* are both associated to the minimum entropy being the unambiguous marks of feminine singular and masculine singular, respectively; and the endings *-e* and *-i*, even if associated to a slightly higher entropy, are mostly used for feminine plural and masculine plural, respectively. Put in different terms, the overall association between inflectional endings and feature values tends to be more transparent and clear-cut in the adjective forms than in the noun forms. If, from a theoretical perspective (see sections 1 and 4.1.3), this may be anything but an unexpected result, nevertheless, it is the first time that these different distributions are quantified and caught in terms of entropy metrics as for the Italian inflectional system.

#### 4.1.2. Transparency of Declensional Classes

When considering declensional classes, the distribution of forms is arranged a little differently across nouns and adjectives. By definition, Italian nouns have a two-cell paradigm, whereas adjectives have a four-cell paradigm (see section 2.1; Iacobini and Thornton, 2016). It follows that the maximum entropy for noun paradigms will be 1 bit, while for adjective paradigms it will be 2 bits. At the same time, entropy allows to quantify information content across paradigms with different numbers of cells. Nonetheless, it is noteworthy that in Italian adjectives information tends to be higher when considering form distribution across feature values, whereas in Italian nouns information grows when considering form distribution across declensional classes.

As for nouns, the most represented classes are *a_e, o_i*, and *e_i*. While the first two classes show the minimum entropy as they almost always host feminine and masculine nouns, respectively, the third class *e_i* shows the close-to-max entropy as masculine and feminine nouns share almost the same probability of being comprised. This is consistent with what is usually stated in the literature, namely that the first two inflectional classes tend to be the most productive as they are more transparent with respect to gender and number features. In other words, newly formed lexical entries are more likely to be assigned to one of the first two Italian declensional classes because these are the most informative ones (Thornton, 2004; D'Achille and Thornton, 2008; Acquaviva, 2009). Even in this case, the overall declensional system seems to reflect a trade-off between maximal discriminability and maximal regularity.

When it comes to adjectives, once again, much less variability is found than for nouns. By far the most represented class, *a_e o_i* is associated to the close-to-max entropy of the distribution since it equally comprises masculine singular, feminine singular, masculine plural, and feminine plural forms.

#### 4.1.3. Form-Value Transparency in Nouns and Adjectives

We suggest that the different distributions of noun and adjective forms we observed so far are related to the distinct functions played by these two parts of speech in agreement relations, in which they usually act, respectively, as controllers and targets. In Italian, the gender and number of nouns are inherent to the lexeme because their encoding is context-autonomous, while the gender and number of adjectives are contextual because their encoding is obligatorily driven by morpho-syntactic agreement (see section 1; for more on inherent vs. contextual inflection: Booij, 1993, 1996). Therefore, since it is the target which is the locus of agreement (Corbett, 2006, p. 12), in the sense that the signpost of agreement surfaces in the form of the target, we expect a more transparent form-value relation in targets than in controllers. Consequently, we expect this to be reflected in their distribution in the language. Word-formation processes in the adjective domain confirm this aspect, with superlative forms in *-issima, -issime, -issimo, -issimi* being assigned to the maximally discriminative class.

To a certain extent, this also applies to adjectives such as *grande-grandi*, “big.SG – big.PL,” in which *-e* is the ending for both masculine and feminine singulars and *-i* is the ending for both masculine and feminine. Although syncretism blurs the gender distinction, the number opposition is still clear-cut. This resonates with general typological trends whereby the feature of number is prioritized over the feature of gender. Indeed, grammatical gender is less widespread across languages (Corbett, 1991) and, as stated in Greenberg's Universal 34, in a language, the presence of number is a necessary condition for gender to surface (Greenberg, 1963) possibly, due to a preminence of the semantic information conveyed by number (Franzon et al., 2019, 2020). Thus, noun forms are less informative with respect to gender (and, to a lesser extent, number) since their main role is to distinguish classes of words mainly favoring discriminability between diverse forms as a whole rather than between gender and number values. Conversely, almost all Italian adjective forms manage to maintain close-to-max discriminability, at least between number values. This result can be interpreted in light of a language processing mechanism; the transparency of targets disambiguates the features of theirs controllers, making their agreement relation explicit. Since targets favor prediction in language processing, it seems reasonable that their form-to-value consistency tends to be more transparent when compared to controllers.

### 4.2. Pending Issues and Conclusions

Our results on Italian nouns and adjectives are compatible with current Word and Paradigm-based approaches from both a theoretical and computational perspective (for an overview see Marzi et al., 2020). However, the differences found in the distributions of nouns (that are generally controllers and have an inherent inflection) and adjectives (that are generally targets and have a contextual inflection) needs to be deepened on. In this respect, while some recent accounts have explored the differences in the effect of syntagmatic and paradigmatic cues in comprehension (Ðurđević and Milin, 2019), only few studies have been dedicated on how contextual and inherent inflection are parsed during language processing (Franzon et al., 2013, 2014). Do the distributional properties measured in this study reflect only mechanisms internal to the morphological organization of (Italian) forms, or are they also a reflection of more general cognitive mechanisms? Literature is scarce in this regard. Despite the fact that consistency between formal cues and gender values has been shown to impact gender retrieval in both isolated word presentation and sentence processing (see, for example, Caffarra et al., 2015), to date no psycholinguistic study has tested whether the observed differences in the distribution of noun forms vs. adjective forms with respect to gender and number values also correspond to differences in processing. Yet, it is possible that inherent inflection and contextual inflection are not merely theoretical constructs. For example, it has been found that, in contact language situations (when a recipient language changes as an effect of contact with a source language), inherent inflection is more likely to be borrowed than contextual inflection since this latter is more entrenched in the grammar and altering it in a resource language causes huge changes in agreement mechanisms. By contrast, the introduction of endings realizing inherent inflection impacts less on the overall morpho-syntactic structure of the recipient language (Gardani, 2012, 2020).

Our results are also consistent with psycholinguistic studies that have related effects on processing to the distributional properties of Italian nouns, reporting slower and less accurate responses to noun forms opaque with respect to gender (De Martino et al., 2011, 2017; Caffarra et al., 2015). Mismatches between declensional class and gender value have been proven costly in processing terms and, in particular, fMRI data showed increased cortical activity for an extensive network (involving frontal and temporal areas, cingulate cortex and cerebellum) linked to inflectional operations for Italian non-transparent declensional classes (Russo et al., 2021). We expect our results to provide a better estimate of Italian nouns' transparency for future neuro- and psycholinguistic studies on inflection.

In this respect, we are well aware that our approach is only one possible way to quantify the regularity of morphological cues. For example, under the umbrella of the competition model, MacWhinney et al. (1984) argued that each mapping between a form and a function can be assigned a weight or strength. The weight of a cue would depend on its validity, i.e., the combination of cue reliability (how many times the cue relates to a specific function) and cue availability (how many times a specific cue is present in the lexicon). We do not comment on the substance of this model. Yet, it is worth noticing that, in the present study, we propose entropy as a measure based on the properties of the signal, as observed in linguistic corpora. To use Mandelbrot's words, three elements are to be considered [for a theory of communication]: (1) The structure of language, or shortly, message; (2) The way in which information is coded by the brain; (3) The economical “criterion of matching” which links 1 and 2 (Mandelbrot, 1953, p. 486). The present work aims at contributing to the knowledge regarding the first element, which is necessary to inform the other two. With this purpose in mind, and with the currently available material, it is not possible to completely disentangle entropy from other linguistic and psycholinguistic variables. However, we believe that this work will nonetheless provide researchers with a useful metric of form-value, that has thus far scarcely been considered (especially with regard to Italian noun and adjective forms), and that this will provide them with a solid ground for the experimental assessment of inflectional morphology-related hypotheses. Moreover, entropy metrics seem to be a suitable and well-grounded tool when comparing typologically diverse languages.

Eventually, although we have measured the entropy of purely morphological systems, the distribution of word forms across inflectional feature values, overall, seems to reflect factors which relate to the morpho-syntactic level and the functions that parts of speech such as nouns and adjectives play at this level. Hence, these entropy metrics are valuable both when testing words in isolation and in sentence context. For all these reasons, we believe that the set of observations we have provided in the present work are potentially relevant for any future study focusing on inflection, in light of the implications that form-value (in)consistency can have for sentence processing, especially with respect to nouns and adjectives. We encourage further research on this topic.

## Data Availability Statement

The datasets presented in this study can be found in online repositories. The names of the repository/repositories and accession number(s) can be found below: https://github.com/franfranz/Flex_it.

## Author Contributions

VP: original idea of the study, manuscript drafting, previous versions of the resource development, and final draft revision. CZ: original idea of the study, manuscript drafting, and final draft revision. DC: scientific supervision to the study and final draft revision. FF: original idea of the study, manuscript drafting, data analysis, development of the current version of the resource, and final draft revision. All authors contributed to the discussion that laid the theoretical foundations of the study and approved the submitted version of the manuscript.

## Funding

This study was supported by H2020 European Research Council [grant number: 679010 (ERC STATLEARN to DC)].

## Conflict of Interest

The authors declare that the research was conducted in the absence of any commercial or financial relationships that could be construed as a potential conflict of interest.

## Publisher's Note

All claims expressed in this article are solely those of the authors and do not necessarily represent those of their affiliated organizations, or those of the publisher, the editors and the reviewers. Any product that may be evaluated in this article, or claim that may be made by its manufacturer, is not guaranteed or endorsed by the publisher.

## Footnotes

^1^$H=-\underset{x}{\sum }p\left(x\right)\log_{2}p\left(x\right)$ where *p*(*x*) is the probability of occurrence of a given word form.
